# Draft Genome Sequence of *Paenibacillus* sp. XY044, a Potential Plant Growth Promoter Isolated from a Tea Plant

**DOI:** 10.1128/genomeA.01016-17

**Published:** 2017-09-14

**Authors:** Huihui Liu, Wenna Shan, Ying Zhou, Xiaomin Yu

**Affiliations:** a Horticultural Biology and Metabolomics Center, Haixia Institute of Science and Technology, Fujian Agriculture and Forestry University, Fuzhou, China; b College of Horticulture, Fujian Agriculture and Forestry University, Fuzhou, China; c College of Life Sciences, Fujian Agriculture and Forestry University, Fuzhou, China

## Abstract

*Paenibacillus* sp. XY044 is an endophytic bacterium isolated from the stem of a tea plant (*Camellia sinensis* cv. Maoxie). Here, we present the draft genome sequence of XY044, which includes genes encoding features related to plant growth promotion and biocontrol.

## GENOME ANNOUNCEMENT

The genus *Paenibacillus* comprises Gram-positive, endospore-forming bacteria ubiquitously occurring in the environment. Many species of this genus function as plant growth promoters, and several have been developed as biological control agents of plant diseases (1). Known to produce a wide range of antimicrobials, hydrolytic enzymes, and exopolysaccharides, *Paenibacillus* spp. have found wide applications in agriculture, medicine, process manufacturing, cosmetics, and bioremediation (2–4). As a result, there is continued interest in genome sequencing of this group of microorganisms to explore their huge potential. Since their first description in 1993, genomes of more than 200 *Paenibacillus* strains representing 82 species have been sequenced (3).

The strain presented here, *Paenibacillus* sp. XY044, is an endophytic strain newly isolated from the stem of a tea plant (*Camellia sinensis* cv. Maoxie) in China. It was cultivated in nutrient broth at 30°C overnight. Genomic DNA was extracted using the DNeasy UltraClean microbial kit (Qiagen, USA) following the manufacturer’s protocol. The draft genome was sequenced using 150-bp paired-end reads on the Illumina HiSeq X Ten system with an average coverage of 188×. A total of 1,402 Mb of clean data for XY044 were assembled into 27 contigs with an *N*_50_ value of 582,877 bp using SOAPdenovo2 software (5). The genome assembly consists of 7,869,445 bp, with a GC content of 52.3%.

Gene annotation was performed using the Rapid Annotations using Subsystems Technology (RAST) server (6). A total of 7,179 putative protein-coding genes and 103 RNA genes were identified. The genome of XY044 presents several genes related to phosphate solubilization and assimilation. For example, XY044 contains genes for glucose-1-dehydrogenase and gluconic acid dehydrogenase, which are involved in the production of gluconic acid, one of the major organic acids responsible for solubilization of inorganic mineral phosphates (7). Genes for the uptake and degradation of phosphonates (organophosphorous molecules with a stable C-P bond), namely, *phn* genes (*phnBEHIJKL*) (8), were also detected in the genome of XY044. In addition, XY044 carries the *pst* operon (*pstS*, *pstC*, *pstA*, and *pstB*) and the PhoP-PhoR system related to P_i_ transport and regulation of P_i_ uptake (9). There also exist in the genome multiple genes encoding siderophores and hydrolytic enzymes, such as chitinases, glucanases, proteases, and cellulases, thus facilitating the inhibition of the growth of fungal pathogens (3, 10, 11).

Overall, the genome sequence of XY044 shows its genetic potential for plant growth promotion and biocontrol, which could potentially be exploited further in the future for biotechnological applications.

### Accession number(s).

This whole-genome shotgun project has been deposited at DDBJ/ENA/GenBank under the accession number NQMO00000000. The version described here is the first version, NQMO01000000.
